# Plasma proteomic changes in response to exercise training are associated with cardiorespiratory fitness adaptations

**DOI:** 10.1172/jci.insight.165867

**Published:** 2023-04-10

**Authors:** Jeremy M. Robbins, Prashant Rao, Shuliang Deng, Michelle J. Keyes, Usman A. Tahir, Daniel H. Katz, Pierre M. Jean Beltran, François Marchildon, Jacob L. Barber, Bennet Peterson, Yan Gao, Adolfo Correa, James G. Wilson, J. Gustav Smith, Paul Cohen, Robert Ross, Claude Bouchard, Mark A. Sarzynski, Robert E. Gerszten

**Affiliations:** 1 Division of Cardiovascular Medicine,; 2 CardioVascular Institute, Beth Israel Deaconess Medical Center, Boston, Massachusetts, USA.; 3 National Heart, Lung, and Blood Institute’s Framingham Heart Study, Framingham, Massachusetts, USA.; 4 Broad Institute of MIT and Harvard, Cambridge, Massachusetts, USA.; 5 Laboratory of Molecular Metabolism, The Rockefeller University, New York, New York, USA.; 6 Department of Exercise Science, Arnold School of Public Health, University of South Carolina, Columbia, South Carolina, USA.; 7 Jackson Heart Study, University of Mississippi Medical Center, Jackson, Mississippi, USA.; 8 The Wallenberg Laboratory, Department of Molecular and Clinical Medicine, Institute of Medicine, Gothenburg University and the Department of Cardiology, Sahlgrenska University Hospital, Gothenburg, Sweden.; 9 Department of Cardiology, Clinical Sciences, Lund University, Lund, Sweden.; 10 Wallenberg Center for Molecular Medicine and; 11 Lund University Diabetes Center, Lund, Sweden.; 12 Department of Heart Failure and Valvular Disease, Skåne University Hospital, Lund, Sweden.; 13 School of Kinesiology and Health Studies, Queen’s University, Kingston, Ontario, Canada.; 14 Human Genomics Laboratory, Pennington Biomedical Research Center, Baton Rouge, Louisiana, USA.

**Keywords:** Cardiology, Metabolism, Cardiovascular disease, Extracellular matrix, Proteomics

## Abstract

Regular exercise leads to widespread salutary effects, and there is increasing recognition that exercise-stimulated circulating proteins can impart health benefits. Despite this, limited data exist regarding the plasma proteomic changes that occur in response to regular exercise. Here, we perform large-scale plasma proteomic profiling in 654 healthy human study participants before and after a supervised, 20-week endurance exercise training intervention. We identify hundreds of circulating proteins that are modulated, many of which are known to be secreted. We highlight proteins involved in angiogenesis, iron homeostasis, and the extracellular matrix, many of which are novel, including training-induced increases in fibroblast activation protein (FAP), a membrane-bound and circulating protein relevant in body-composition homeostasis. We relate protein changes to training-induced maximal oxygen uptake adaptations and validate our top findings in an external exercise cohort. Furthermore, we show that FAP is positively associated with survival in 3 separate, population-based cohorts.

## Graphical abstract

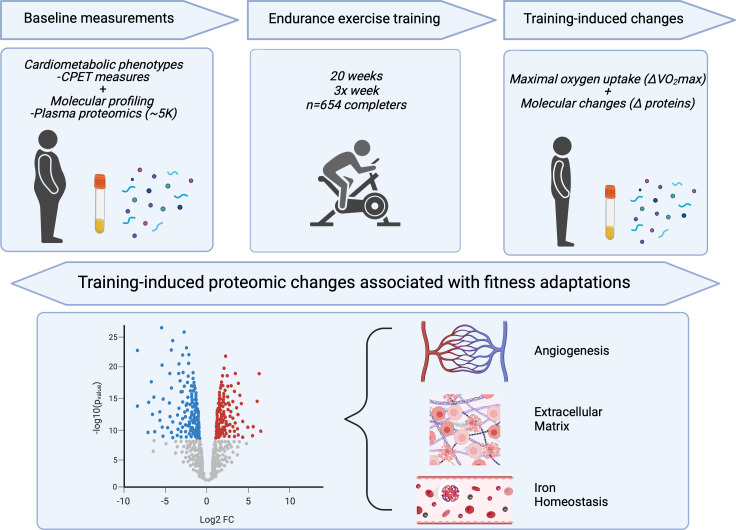

## Introduction

The beneficial health effects of regular exercise are many, spanning the cardiopulmonary, endocrine, and nervous systems, among others. Physiologic adaptations to regular exercise reflect a highly coordinated, multiorgan-system effort. Although substantial progress has been made toward understanding these adaptations at the molecular and cellular levels (1, 2), particularly in the skeletal muscle (3, 4), much remains to be understood about the body’s global and integrative molecular responses to exercise. Indeed, unraveling the biochemical pathways behind exercise-induced health adaptations has emerged as an important goal of the medical community (5).

Plasma proteins play an important role in cardiovascular and metabolic health, both as effectors of physiologic processes (6, 7) and predictors of health outcomes (8, 9). A growing body of data has demonstrated that individual circulating proteins are modulated by exercise and may transduce health benefits (10–13). Prior efforts to characterize proteomic responses to exercise stimuli have focused on skeletal muscle changes (14) and posttranslational modifications after single bouts (15) or have been limited in size or scale. These and other data (4) have helped describe the kinetic transcriptional and protein responses to acute exercise; however, little is known about the global, circulating protein changes that occur in response to chronic exercise in humans.

We previously described plasma proteins associated with maximal oxygen uptake (VO_2_max) in the untrained (baseline) state in cross-sectional analyses (16). By contrast, here we leverage new, postexercise-training plasma proteomic data to characterize the protein changes that occur in response to regular endurance exercise in 654 participants before and after a 20-week training program. We hypothesized that relating training-induced plasma proteomic changes to the changes in VO_2_max would highlight individual molecules and networks involved in cardiorespiratory fitness (CRF) adaptation. Given the well-established relationship between CRF and mortality, we then sought to examine the association between candidate proteins from these exercise-training analyses and longitudinal health outcomes across multiple population-based cohorts.

## Results

### Plasma proteomic changes after endurance exercise training in HERITAGE.

The mean (SD) age of the Health Risk Factors, Exercise Training, and Genetics (HERITAGE) cohort was 35 (13.6) years. Of the 654 participants, 230 were Black (35%) and 361 were women (55%). The mean (±SD) VO_2_max prior to exercise training was 2348 (±733) mL/min and the change in VO_2_max (ΔVO_2_max) was 383 (±203) mL/min (Table 1).

**Figure 1:**
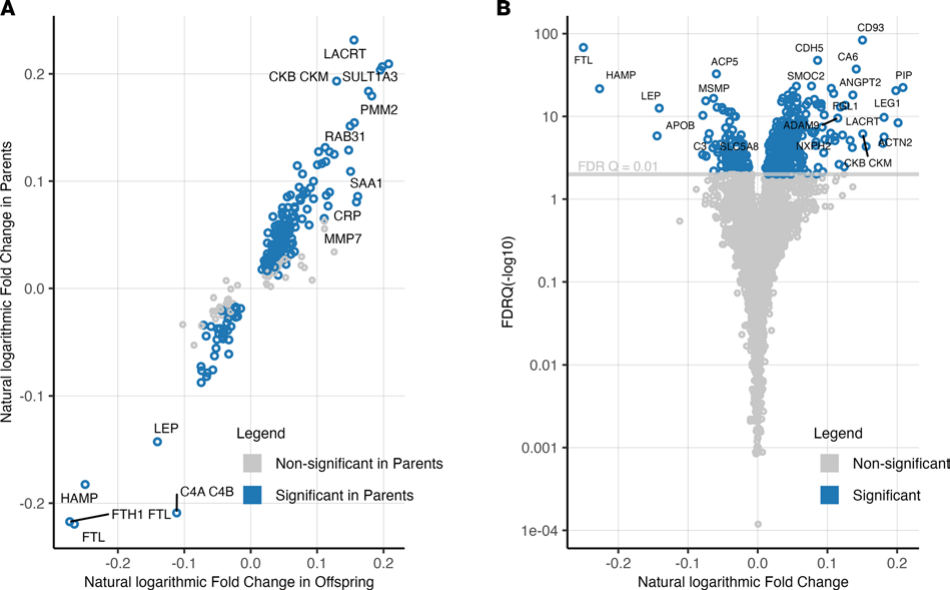
Plasma protein changes after 20 weeks of endurance exercise training. Log_10_ transformed protein changes after exercise testing were assessed in a paired Student’s *t* test. (**A**) Proteins that changed after exercise training (FDR < 0.01) in the offspring subgroup (internal derivation cohort; *x* axis) were tested in the subgroup of parents (internal validation cohort; *y* axis). Protein levels that increased after exercise are shown in the right quadrants (offspring) and top quadrants (parents). Proteins that decreased after training are located in the left and bottom quadrants, respectively. (**B**) Protein FCs after exercise training in the full cohort. A total of 453 proteins changed at FDR < 0.01.

We assayed 4914 unique protein targets according to gene ontology (GO) annotation. As previously described (16), we first analyzed the parents (*n* = 221) and offspring (*n* = 443) in the HERITAGE cohort separately to evaluate the consistency of our findings (Table 1). We found that the levels of 245 of proteins changed (FDR *q* < 0.01) after exercise training in the larger, offspring subgroup, and 184 of those proteins (75%) also changed (*P* < 0.05) in the parents (Figure 1). Given the consistency of findings across generations, we subsequently used the full cohort for further analyses.

In the full HERITAGE cohort, the levels of 453 proteins changed after exercise training (*q* < 0.01) (Figure 1 and Supplemental Table 1; supplemental material available online with this article; https://doi.org/10.1172/jci.insight.165867DS1). Of these, the levels of 306 proteins (68%) increased after exercise training, approximately 40% of which constituted secreted proteins according to UniProt Consortium data (Supplemental Figure 1) (17). To support the specificity of our proteomic findings, we performed additional profiling using Olink’s Ab-based platform in a representative subsample of 209 HERITAGE participants, and we also used proteo-genetic information (6, 7, 18–23) to test whether plasma protein levels are associated with genetic variants in their respective cognate genes (i.e., cis protein quantitative trait loci). Among the 25 proteins with the most significant changes after exercise training that overlapped the Olink platform, 21 aptamer-based assays had either a Spearman correlation (ρ) greater than 0.5 with its respective Olink counterpart (median ρ, 0.63 [95% CI, 0.53–0.70]) (Supplemental Table 2) and/or cis variants associated with its circulating protein level (Supplemental Table 3).

We identified protein changes among both expected and unanticipated biological pathways. For example, we found several circulating proteins related to angiogenesis, including increases in VEGF-signaling pathways (i.e., VEGFA and both neuropilins [NRP] 1 and 2), vascular homeostasis (i.e., angiopoietin-2 [ANGPT2]), and proteins involved in capillary basement membrane turnover (i.e., MMP19). Although these biologic systems have been associated with regular exercise in human or mouse models, many individual proteins have not been described in the circulation. Similarly, we found decreases in hepcidin concentrations and increases in concentrations of hemojuvelin, a hepcidin regulator, in addition to previously described exercise-induced changes in iron homeostatic (decreased ferritin [FTL]) and erythropoietic factors (increased erythropoietin) (24).

### Relationship between protein changes and BW and composition changes.

Given that the plasma proteome may reflect proteins derived from numerous tissue sources, including adipose and/or skeletal muscle tissue, we examined the relationships between body weight (BW) and composition changes and protein changes. Baseline measures of total BW, lean body mass, and body fat percentage and their changes after exercise training are summarized in Supplemental Table 4. Protein changes (*n* = 453) were weakly correlated with total BW changes (median *r* = 0.04; range, –0.194 to 0.276), lean body mass (*r* = 0.007; range, –0.12 to 0.16), and body fat percentage: (*r* = 0.007; range, –0.193 to 0.15) (Supplemental Table 5).

**Figure 2:**
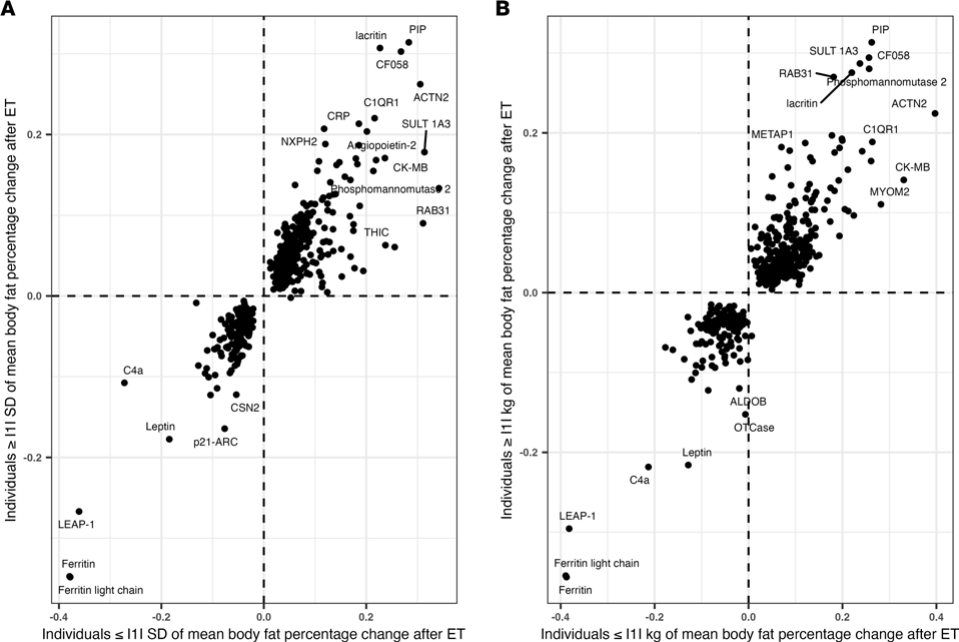
Protein changes according to BW and composition changes after exercise training (ET). (**A** and **B**) Log_10_ protein FCs (*x* and *y* axes) among HERITAGE participants according to BW changes greater or less than 1 kg after exercise training and body fat percentage changes greater or less than 1 SD of the mean after exercise training.

Although the BW changes that occurred after exercise training in HERITAGE were small by design, interindividual heterogeneity of both BW and composition existed and has been previously described (25). Thus, we performed subgroup analyses to test whether the plasma proteomic changes identified in our full cohort differed among participants who experienced small or more substantial changes in BW and composition. Both the magnitude and direction of the protein changes were similar among HERITAGE participants who experienced not more than versus greater than a 1 kg change in BW after exercise training (Figure 2A) and among those who experienced not more than versus greater than a 1 SD of the mean (0.8% ± 1.9%) body fat percentage change (Figure 2B).

### Extracellular matrix proteins are overrepresented among proteomic changes.

**Figure 3:**
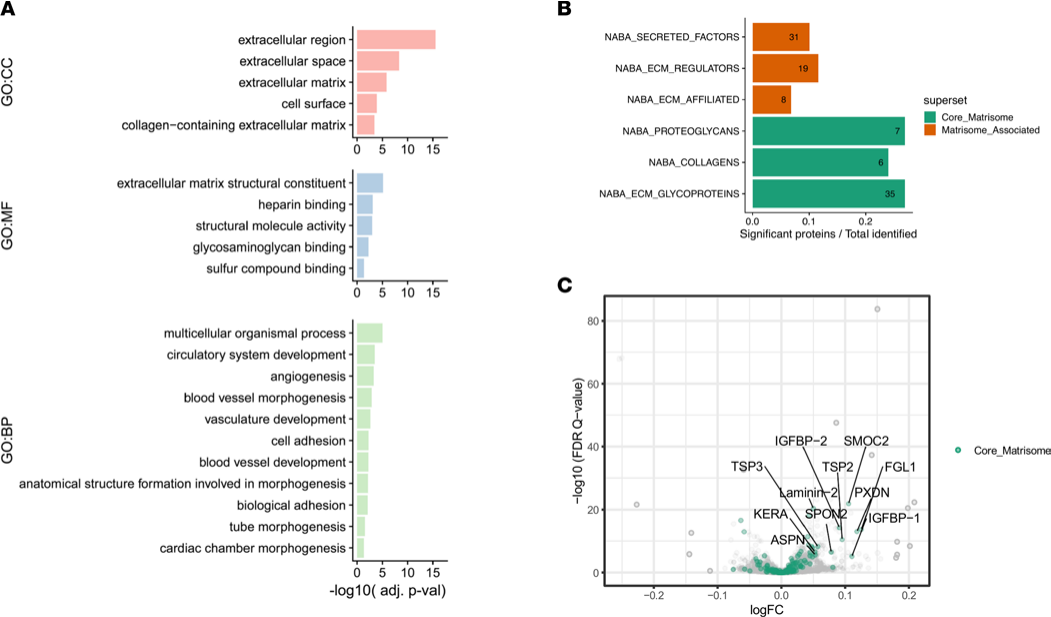
ECM and ECM-related proteins are modulated by endurance exercise training. (**A**) Pathway enrichment analysis of plasma proteins that change after exercise training using GO annotation. The enrichment was performed with proteins that increased (log FC > 0; FDR *q* < 0.01) with exercise training. (**B**) Proteomic changes according to ECM and ECM-related proteins. (**C**) The majority of changed proteins fell into core “matrisomal” groups (individual proteins highlighted in green shown in **C**). Adj. p-val, adjusted *P* value; BP, biological process; CC, cellular component; MF, molecular function.

We applied enrichment analyses to detect overrepresented biological pathways and processes according to GO hierarchy and across sub-ontologies (26). Extracellular matrix (ECM) and ECM-related proteins, many of which exist as membrane-tethered and/or secreted factors, were among the most overrepresented features (Supplemental Table 6). Additional analyses using a curated list of ECM-secreted factors, matrisome, and matrisome-related proteins according to MatrisomeDB (http://matrisomedb.pepchem.org/) (27) confirmed enrichment of matrisomal (i.e., collagens, glycoproteins, and proteoglycans) and matrisome-related protein changes after training (Figure 3). In total, we identified 62 proteins previously annotated as ECM factors (27, 28), the majority of which (76%) increased after exercise training (Supplemental Table 1). Using published skeletal muscle expression data from the Meta-Analysis of Skeletal Muscle Response to Exercise (MetaMEx) database of human aerobic exercise training studies (*n* = 256–337 individuals; https://www.metamex.eu/) (29), we found directionally consistent transcriptomic changes among 41 of the 62 ECM protein targets. Additional pathways related to vasculo- and angiogenesis were also enriched in exercise training (Supplemental Table 6).

### Protein changes specifically associated with VO_2_max adaptation.

We next examined the relationship between the change in proteins and ΔVO_2_max and, again, found overrepresentation of the ECM in gene set enrichment analysis (GSEA) (Supplemental Figure 2). Individually, the absolute changes in fibroblast activation protein (FAP), ANGPT2, cadherin-5 (CDH5), FTL, and prolargin (PRELP) were associated with ΔVO_2_max in a linear regression model adjusted for age, sex, race, BMI, and baseline (pretraining) VO_2_max. We further adjusted for lean body mass, given previously described differences in protein–VO_2_max relationships according to body composition adjustments (Table 2) (16). Although PRELP and ANGPT2’s relationships were attenuated and no longer statistically significant, the other proteins remained highly associated with ΔVO_2_max.

### Validation of protein findings in an external exercise cohort.

To examine the generalizability of our findings, we measured plasma proteins before and after 24 weeks of endurance exercise training in an external cohort of abdominally obese (waist circumference >88 and 102 cm for women and men, respectively) individuals who participated in an exercise dose-response trial (30). A total of 162 participants completed exercise training in the validation cohort; we restricted analyses to individuals with a baseline BMI of not greater than 35 kg/m^2^ (*n* = 113) to more closely approximate the clinical characteristics of HERITAGE participants, who were nonobese. The validation cohort was older (mean age, 53 years) and had greater body mass (median BMI, 31) than HERITAGE participants (Supplemental Table 7). Among the 5 proteins related to ΔVO_2_max in HERITAGE, all demonstrated directionally consistent and statistically significant changes in the validation cohort. Among the top 25 greatest protein changes in HERITAGE available in the validation cohort, 15 reached at least nominal (*P* < 0.05) statistical significance with the same direction of effect (Supplemental Table 8). Overall, 352 of the 453 protein findings from HERITAGE were available in the validation cohort; 199 demonstrated directionally consistent changes and 84 were statistically significant for these changes (*P* < 0.05).

### FAP protein is positively associated with baseline VO_2_max and a secreted protein.

We next tested the change in protein–ΔVO_2_max findings (i.e., for FAP, ANGPT2, CDH5, FTL, and PRELP) for their baseline (pretraining) relationship with baseline VO_2_max. FAP and CDH5 levels were positively associated with baseline VO_2_max (mL O_2_/min) at nominal (*P* < 0.05) significance after adjusting for age, sex, race, and BMI (β = 30.2 and 29.4, respectively; and *P* = 0.04 and 0.04, respectively). No other proteins were significantly associated with baseline VO_2_max (for ANGPT2, FTL, and PRELP: β = 11.5, –10.1, and 17.1, respectively; *P* = 0.41, 0.55, and 0.23, respectively).

CDH5 (vascular-endothelial cadherin) is a well-described transmembrane protein that helps maintain the endothelial adherens junction and may play an important role in angiogenic sprouting (31); however, it has no known biological activity in circulation. In contrast, FAP is a serine protease involved in ECM remodeling that exists as both a membrane-bound protein and an enzymatically active, secreted protein with relatively unknown circulating function. Thus, we further investigated FAP.

Prior studies have suggested a role for FAP in adipocyte differentiation and BW, as well as maintenance of muscle mass, in model systems (32, 33). To test the influence of body mass and composition on FAP’s relationship with VO_2_max, we compared BMI, body fat percentage, and lean body mass across quartiles of FAP levels. Baseline BMI levels were similar across quartiles of FAP, whereas baseline body fat and lean body mass were inversely and positively associated with increasing quartile of FAP, respectively (Supplemental Figure 3). We subsequently adjusted our baseline VO_2_max analyses for body fat and lean body mass and found that FAP’s positive relationship remained significant (Table 3). We next adjusted our ΔFAP–ΔVO_2_max analyses for changes in body fat (Table 2, model 2) and lean body mass (model 3) after training and found that, despite FAP’s relationship to these traits, ΔFAP remained positively associated with VO_2_max adaptation.

### FAP levels are correlated with striated muscle-specific proteins in the circulation and skeletal muscle expression increases after regular exercise in a murine model.

To begin to understand circulating FAP’s biology, we used the full plasma proteomic platform in HERITAGE and found that circulating proteins related to striated muscle were overrepresented among the top 100 associations with FAP, according to the TISSUES expression database (34). FAP was positively correlated with myostatin (ρ = 0.39; *P* = 1.0 × 10^–28^), creatinine kinase M-type (ρ = 0.33; *P* = 1.3 × 10^–20^), and troponin-I (ρ = 0.31; *P* = 8.6 × 10^–19^) (Supplemental Table 9).

**Figure 4:**
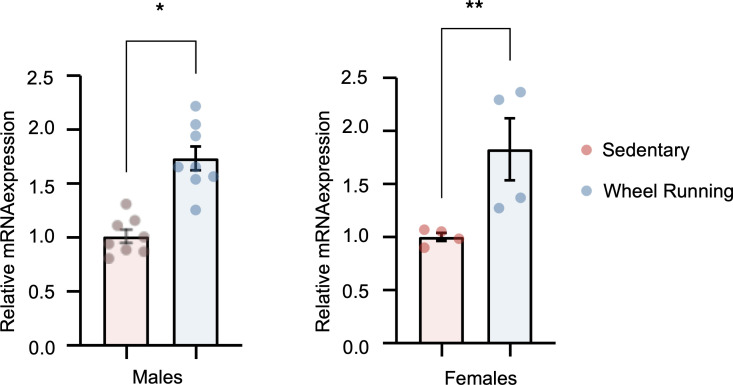
*Fap* skeletal muscle mRNA expression in C57BL/6J mice undergoing voluntary wheel running compared with sedentary controls. mRNA expression in gastrocnemius tissue among male and female sedentary mice and those undergoing voluntary wheel running after 2 weeks (*n* = 8 per group for male mice and *n* = 4 per group for female mice). The data are shown as mean ± SEM. *P < 0.0001, **P < 0.05. Two-tailed unpaired *t* test.

We subsequently tested whether *Fap* expression was regulated in murine skeletal muscle in response to regular exercise. Using a voluntary wheel-running system in C57BL/6J mice, we found differential expression of *Fap* in gastrocnemius muscle of exercising male and female mice in comparison with sedentary control mice (*n* = 8 and 4 per group, respectively) after 2 weeks of wheel running (fold-change [FC], 1.7 and 2.3, respectively; *P* < 0.0001 and 0.05, respectively) (Figure 4).

### FAP levels are inversely associated with all-cause mortality in the Framingham Heart Study, Malmö Diet and Cancer Study, and Jackson Heart Study.

Given the well-established relationship between CRF and mortality, we next tested whether circulating FAP levels were, in turn, associated with all-cause mortality across population-based cohorts of different ancestry. We included a broad set of demographic and clinical covariates based on a priori relationships with long-term health outcomes, including age, sex, BMI, smoking status, systolic blood pressure, estimated glomerular filtration rate, fasting plasma glucose level, and prevalent diabetes, cardiovascular disease (CVD), and cancer (available in Framingham Heart Study [FHS] and Malmö Diet and Cancer Study [MDCS] data only; refs. 35–38).

**Figure 5:**
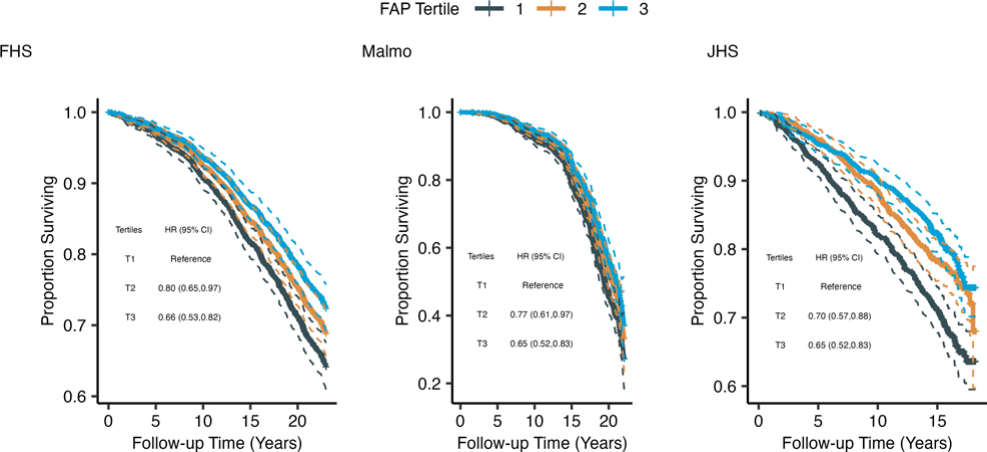
Kaplan–Meier survival analysis according to FAP levels in FHS, MDCS, and JHS. Kaplan–Meier curves displaying time to death by FAP tertile among FHS, MDCS, and JHS cohort participants.

In the FHS, MDCS, and Jackson Heart Study (JHS), 1909, 1324, and 2146 participants, respectively, were included in analyses; clinical characteristics are summarized in Supplemental Table 10. A total of 551, 401, and 557 participants in the respective studies died after a mean (SD) follow-up of 13.6 (5.6), 13.2 (4.2) and 9.5 (4.5) years, respectively. The risk of death decreased with increasing tertile of FAP level across all cohorts (Figure 5). Analyzing FAP level as a continuous variable in FHS, MDCS, and JHS revealed a 16% (95% CI, 7%–24%; *P* = 0.0005), 10% (95% CI, 1%–11%; *P* = 0.04), and 19% (95% CI, 11%–27%; *P* = 0.00003) decrease in all-cause mortality risk per 1 SD increase in FAP, respectively.

To test whether FAP levels provide information in addition that provided by existing biomarkers, we included log-transformed B-type natriuretic peptide (BNP) levels, a well-validated protein biomarker of all-cause mortality (39), in our model. FAP remained a significant predictor of all-cause mortality among all 3 cohorts (for FHS: HR, 0.85 [95% CI, 0.74–0.97]; for MDCS: HR, 0.90 [95% CI, 0.81–0.99]; for JHS: HR, 0.81 [95% CI, 0.73–0.89]). Of note, BNP and FAP levels were poorly correlated (*r* = –0.09, –0.08, and –0.12 for FHS, MDCS, and JHS, respectively).

We further examined the relationship among FAP levels, CRF, and mortality in FHS, where we were able to calculate estimated CRF (eCRF) from a published, non–exercise-based equation (40). Mean eCRF values for men and women were 11.1 and 8.7 metabolic equivalents, respectively. eCRF was inversely associated with mortality in Cox proportional hazards regression in a minimally adjusted model (HR, 0.83 [95% CI, 0.79–0.89], adjusted for age, sex, and proteomics batch) as well as our fully adjusted model (HR, 0.69 [95% CI, 0.6–0.8], adjusted for age, sex, proteomics batch, BMI, smoking status, systolic blood pressure, estimated glomerular filtration rate, fasting plasma glucose level, and prevalent diabetes, CVD, and cancer). The addition of FAP levels to each model significantly decreased the strength of relationship between eCRF and mortality (HR, 0.85 [95% CI, 0.8–0.91] and 0.71 [95% CI, 0.61–0.81], respectively; likelihood ratio test, χ^2^ = 30.4 and 12.0, respectively; *P* = 3.4 × 10^–8^ and 5.2 × 10^–3^, respectively).

## Discussion

The paradigm of exercise-stimulated circulating proteins leading to beneficial health effects has long been established; however, emerging, large-scale proteomics technologies have rapidly expanded our capacity to identify new molecules and pathways that may be involved in this process. Here, we measured approximately 5000 proteins in 654 sedentary human study participants free of cardiometabolic disease before and after an endurance exercise training program to demonstrate broad plasma proteomic changes and provide new insights into CRF adaptation by relating them with VO_2_max changes and subsequent mortality risk.

To our knowledge, this is the first study that characterizes large-scale plasma protein changes after an exercise training intervention in a large human population. We found that approximately 10% of the circulating proteins on our platform were modified by training. To ensure sufficient washout from the effects of acute exercise and capture the effects of a chronic exercise stimulus, we collected plasma during fasting and resting conditions prior to and at least 24 hours after the final exercise training session. Approximately 40% of our plasma protein findings were secreted factors, including several established hormones (i.e., leptin, N-terminal pro-BNP, and follistatin-related protein 1) as well as additional proteins that act in autocrine and paracrine fashions (e.g., chymotrypsin-like elastase family member 2a, tissue inhibitor of metalloproteinases 1, MMP19). This class of proteins is of particular interest given the increasing recognition of exercise-secreted bioactive factors (i.e., “exerkines”) involved in human health (41). Although our findings are limited to association, we highlight new circulating biology that recapitulates tissue-specific health benefits and may be relevant in exercise adaptation.

Previous studies have shown that exercise training leads to increased mRNA expression and protein translation of angiogenic factors at the skeletal muscle level (42); however, limited data exist regarding the effects of chronic exercise on circulating factors (43, 44). We add to this body of work by demonstrating enrichment in the blood for several members of this process, including VEGF-A, NRP1, NRP2, TIE1, and ANGPT2. Both VEGFA and NRP1 levels increase after exercise training; these are factors that are necessary for coronary artery collateral vasodilation (45, 46). Furthermore, ANGPT2 is a secreted growth factor that mediates its effects on angiogenesis through the tyrosine-protein kinase receptor Tie-2 (TIE2) and whose levels increased significantly after exercise training among HERITAGE participants. ANGPT2 exerts context-dependent effects on angiogenesis according to several conditions, including endothelial cell stress (47), ANGPT1 levels (48), and TIE-2/integrin expression (49). Here, we found a nonsignificant decrease in ANGPT1 levels after exercise training. Although speculative, our findings that increased ANGPT2 levels are associated with improvements in VO_2_max could be seen in the context of a proangiogenic response after a chronic exercise intervention. Research on the systemic effects of circulating angiogenic factors is warranted.

We further identified potential exercise-responsive proteins involved in CRF adaptation through the discovery that FAP, a membrane-bound protein that is highly expressed during tissue remodeling (50) but also exists as a classically secreted factor (51), increases after exercise training and is positively related to VO_2_max gains. FAP is a member of the dipeptidyl peptidase-4 activity and/or structural homologue protein family (52). Despite its similarities, its circulating function remains unknown. Our work demonstrating plasma FAP level’s positive relationship with baseline and VO_2_max training adaptations despite adjustment for body mass and composition suggests that the plasma FAP level relationship to fitness may proceed independent of adiposity. Although we are unable to identify the tissue origin of the plasma proteins assayed, we highlight FAP’s relationship to circulating proteins relevant in striated muscle biology and its increased expression in skeletal muscle in a murine model of regular physical activity as a potential site of origin. Indeed, these findings are consistent from MetaMEx human skeletal muscle expression data after aerobic exercise training (29).

Our human data demonstrating circulating FAP’s inverse association with all-cause mortality raise questions about FAP’s role as simply a biomarker of fitness or a physiologically relevant protein in its soluble form, though the small but significant attenuation in eCRF’s inverse association with mortality after adjusting for FAP lends support to the latter possibility. Although pharmacologic inhibition of FAP in resting mice did not confer a major metabolic phenotype (53), prior work has demonstrated that differential protein expression can lead to an exercise-training response but not a resting phenotype (54). Furthermore, mechanistic studies will be essential to help determine whether circulating FAP is involved in exercise adaptation.

Our FAP results also highlight a main finding from our data: the breadth of ECM and ECM-related plasma protein changes that occur after exercise training. Prior efforts have detailed increased ECM tissue expression in response to training and subsequent skeletal muscle adaptation (55, 56); however, its role in remodeling is not well understood and little information exists about these factors in circulation. Furthermore, few proteomic studies of the ECM’s role in cardiometabolic health exist, and those that do have focused on disease states (57, 58) or been applied in non–plasma-based samples (59). Here, we describe a broad array of proteins that span both the structural and nonstructural ECM, including proteoglycans (e.g., perlecan, biglycan, thrombospondins 2–4), collagens (e.g., collagen alpha-1 [III], [XV], and [XXVIII]), and laminins, as well as enzymes involved in ECM turnover and remodeling (e.g., MMP19, TIMP-1, disintegrin and metalloproteinase domain-containing protein 9, and FAP), that change in circulation in response to exercise training.

Several possibilities exist to explain these findings. First, as previously described, the ECM and, in particular, the intramuscular connective tissue and its resident adult muscle stem cell (“satellite cell”) microenvironment, is dynamically modified by physical activity and during skeletal muscle repair and adipose tissue remodeling (60–62). It is possible that small but meaningful changes in ECM tissue sources (i.e., adipose and skeletal muscle) after exercise training contribute to circulating ECM levels, although we cannot exclude contributions from additional tissues (i.e., myocardium) that remodel with endurance exercise. We again note that we were unable to identify the tissue origin of the plasma proteins on this platform.

Second, there is increasing recognition that circulating ECM factors may be functionally important in skeletal muscle. For instance, the small-leucine rich proteoglycan biglycan regulates the expression of utrophin in myotubes as well as in murine sarcolemma, and systemic delivery of recombinant human biglycan decreased fibrosis in a murine model (*mdx* model) of Duchenne muscular dystrophy (63). Indeed, biglycan is currently under investigation as a therapy for Duchenne muscular dystrophy (64). We found significant increases in circulating biglycan (log FC = 0.045; FDR *q* = 0.0018) in HERITAGE participants after exercise training. These findings, in addition to increased FAP expression in murine and human skeletal muscle and in human plasma after regular exercise, raise the possibility that regular exercise promotes favorable skeletal muscle remodeling via the release of circulating ECM factors. This, in turn, may improve systemic oxidative metabolism and CRF. Indeed, the ECM may have unanticipated roles in cardiovascular biology (65, 66). We add to these emerging data by highlighting the influence of exercise training on plasma levels of this broad system and support further study regarding the mechanistic function of individual candidate proteins.

### Study limitations.

We did not use an effect-size threshold to determine proteins that changed after exercise training, thus possibly decreasing the specificity of our findings. No large-scale plasma proteomic profiling of exercise training in humans exists, to our knowledge; thus, limited prior information exists to guide what effect sizes represent biologically meaningful changes among many individual proteins and after exercise training. We chose, instead, to use a more conservative statistical threshold (FDR < 1%) to determine significance.

We validated our top protein findings, including FAP and the additional change in protein–ΔVO_2_max findings, in an external cohort; however, discordant findings existed among other proteins. We believe that large differences in clinical characteristics may have contributed. For instance, participants in the validation cohort lost a median of 4.5 kg after training in contrast to HERITAGE participants, who maintained weight neutrality; here, we found several discordant protein findings that may be influenced by weight changes (e.g., C-reactive protein, adiponectin). Additional replication of our HERITAGE findings will be needed in a large, healthy adult cohort such as the NIH’s Molecular Transducers of Physical Activity Consortium (MoTrPAC) Study (5). In the interim, all our proteomics data have been made publicly available to the scientific community for further investigation. HERITAGE is a single-arm exercise-intervention study, and although repeated cardiopulmonary exercise testing (CPET) measures both before and after training were performed and demonstrated low variability in measures of CRF (VO_2_max coefficient of variation [CV], 5%), we cannot exclude additional factors that contribute to posttraining changes.

In summary, our study details large-scale plasma proteins modulated by an endurance exercise training program and highlights the ECM among this group. We identify circulating FAP as an exercise-responsive protein positively related to CRF and long-term health outcomes that warrants additional study into its mechanistic function.

## Methods

### HERITAGE Family Study

#### Participants.

A total of 654 individuals from families of European and African descent (38% African ancestry; age range, 17–65 years) were recruited and completed a 20-week, graded endurance exercise training program across 4 clinical centers in the United States and Canada between 1993 and 1997. Participants were healthy but sedentary over the previous 3 months, and free from cardiometabolic disease. The 654 participants completed exercise training and underwent complete CPET and plasma sampling. Written informed consent was obtained from all HERITAGE participants. HERITAGE study consent was reviewed and the research performed in this analysis was approved by Beth Israel Deaconess Medical Center’s IRB.

#### CPET protocol.

Two separate maximal exercise tests were performed on separate days, at least 48 hours apart, before and after the 20-week training program using a cycle ergometer (model 800S; SensorMedics) connected to a metabolic cart (model 2900; SensorMedics). Standard gas-exchange measures were obtained as an average of 20-second intervals. VO_2_max criteria were defined as a respiratory exchange ratio greater than 1.1, a plateau in VO_2_ uptake (i.e., a change of <100 mL/min in the last 3 consecutive 20-second averages), and an heart rate within 10 beats/min of the maximal level predicted by age. All participants met the criteria for at least 1 of the maximal tests. The average of the 2 measurements before and after exercise training was used as the VO_2_max unless the values differed by more than 5%, in which case the higher value was used. The correlation between VO_2_max measurements between the 2 tests (*r* = 0.97), CVs (5%), and reproducibility among clinical centers were excellent (67).

#### Body mass and composition.

Body mass was measured to the nearest 0.1 kg using a balance beam scale. Body composition was assessed using hydrostatic weighing. Residual lung volume was assessed out of water using the oxygen-dilution principle at 3 clinical centers and using a helium-dilution method at the fourth center. The average of the 2 highest measures taken on 3 separate days for both sets of traits was used and demonstrated excellent CVs and reproducibility (68).

#### Exercise-training protocol.

Participants exercised 3 times per week for 20 weeks under full supervision, beginning at 30 min/session and increasing to 50 min/session for the final 6 weeks of the program. Exercise intensity increased from the heart rate associated with 55% VO_2_max obtained during baseline CPET to the heart rate associated with 75% VO_2_max over the final 8 weeks of training. Cycle ergometers (Universal Aerobicycles) were electronically programmed to maintain the prescribed training heart rate by adjusting the power output (69). Participants included in this study completed at least 95% of the training program.

#### Plasma sampling in HERITAGE.

Plasma samples were collected, via peripheral i.v. catheters into EDTA tubes, from participants at rest before beginning the exercise training program, and then again at least 24 hours after completing the last session to avoid contamination from the last acute exercise bout and reflect the adaptive changes of chronic training. Plasma was stored at –80°C and underwent either 0 or 1 freeze-thaw cycle prior to proteomics profiling.

### Population-based cohorts

#### FHS.

FHS is a multigenerational, population-based prospective cohort study originally designed to understand the epidemiology of coronary heart disease in the United States. Here, we included a total of 1909 participants from the FHS Offspring Cohort who attended the fifth examination (1991–1995) and previously underwent plasma proteomic profiling using the SOMAscan platform (35). Proteomics data were acquired from 1835 participants across 2 batches. All-cause mortality data were obtained from hospital admission and emergency department records; physician notes; death certificates; or, in the absence of other records, a telephone call to family members by a physician. Events were adjudicated by a panel of 3 physicians to further determine cause of death (70). All participants provided written informed consent and were approved by the IRBs at Boston University Medical Center and Beth Israel Deaconess Medical Center.

#### MDCS.

The MDCS is a Swedish, prospective case-control study originally designed to investigate the effects of diet and genetic variation on cancer and other long-term health outcomes (36). A total of 1394 participants who underwent plasma proteomic profiling were included in analyses (9). All-cause mortality data were obtained from the Swedish National Death Registry and the National Tax Board using individual civil registration numbers. All participants provided written informed consent and analyses were approved by local ethics committee (Lund University) as well as the IRB of Beth Israel Deaconess Medical Center.

#### JHS.

The JHS is a prospective, population-based, observational study of individuals from the Jackson, Mississippi, metropolitan area, designed to study risk factors for CVD in Black adults. The JHS study design, recruitment, and data collection, including mortality ascertainment, have been described previously (37, 38). The JHS was approved by the IRBs of Jackson State University, Tougaloo College, and the University of Mississippi Medical Center in Jackson, Mississippi. All study participants provided written informed consent. The current analysis was also approved by the IRB of Beth Israel Deaconess Medical Center.

#### Queen’s University Study.

The Queen’s University study design has been described (30). Briefly, 162 abdominally obese participants completed 24 weeks of endurance exercise training and underwent both cardiopulmonary exercise training and plasma sampling before and after completion. Proteomics profiling was performed using an earlier version of the aptamer-based, SOMAscan platform (SomaLogic Inc.) described below.

### Proteomics profiling

#### Aptamer-based proteomics profiling.

Briefly, stored plasma samples were diluted in 3 different concentrations (40%, 1%, and 0.05%) and subsequently incubated using aptamer reagents: fluorescently labeled ssDNA chemically modified to enhance epitope binding. The assay measures plasma proteins using a multistep capture, release, and recapture enrichment process. Streptavidin bead–immobilized aptamers bind plasma proteins that are then biotinylated. Aptamer-bound proteins are then photocleaved, released, and quantified using a DNA oligo-array plate reader that provides relative fluorescent units. Samples are run in 96-well plates that include 11 wells used for quality control. Five samples from external pooled human-plasma specimens are used to assess intra-assay CVs, and 3 pooled plasma samples from within experiments are used to calculate inter-assay CVs. Sample data were median-normalized and calibrated to remove assay differences between runs. In HERITAGE, the approximately 5000-plex SOMAscan platform was used, whereas in FHS, MCDS, and JHS, an approximately 1100- to 1300-plex platform was used. In the Queen’s University Study, an approximately 4000-plex (earlier versions) of the platform was used.

#### Ab-based proteomics profiling.

We subsequently performed proteomics profiling using Ab-based technology (Olink) on a random sample (*n* = 209) from the HERITAGE study to determine the reproducibility of our aptamer-based results. Briefly, the Olink plasma-extension assay technology uses DNA oligonucleotide–labeled Ab pairs to bind target proteins. A total of 384 assays are performed on 4 separate panels with different dilutions for different dynamic ranges of target proteins (*n* = 1536 total proteins assayed). After incubation with plasma samples, the oligonucleotide pairs hybridize and are extended by DNA polymerase to create a unique DNA barcode that is subsequently read out using next-generation sequencing (Illumina NovaSeq). The assay readout is presented in Normalized Protein eXpression values, an arbitrary, relative quantitative unit in log_2_ scale; higher values correspond to higher protein expression. Additional data regarding the platform’s limit of detection and precision are available on the manufacturer’s website (www.olinkexplore.com). The median intra- and inter-assay CVs across the platform were 11.25% and 17.5%, as assessed by multiple replicates of a pooled plasma sample included in each plate.

### Mouse studies

Male and female C57BL/6J mice (The Jackson Laboratory; RRID: IMSR_JAX:000664) were provided food and water ad libitum with standard rodent diet (Labdiet Picolab Rodent, 5053). Mouse experiments were performed in an animal facility maintained at 22°C and 50% humidity level, with a 12-hour light cycle. For the voluntary wheel-running intervention, mice were housed individually and had free access to voluntary wheel running (Columbus Instruments 0297-0521). Beginning at 16 weeks of age, mice performed wheel running for 2 weeks. The mean daily running distance was 7.1 km and 4.1 km for female and male mice, respectively. Mouse studies were performed in accordance with the institutional guidelines of The Rockefeller University IACUC (protocol 18016-H).

### RNA purification, cDNA synthesis, and real-time quantitative PCR

At 18 weeks of age, mice were euthanized by isoflurane overdose, the gastrocnemius muscle was dissected using surgical tools and then immediately frozen in liquid nitrogen and stored at –80°C until further processing. For purification of RNA, the gastrocnemius muscle was resuspended Trizol reagent (Invitrogen; catalog 15596026) and homogenized mechanically, and RNA was separated with chloroform (Fisher Scientific; catalog C298) and purified on a silica column (RNeasy mini kit, catalog 74104; Qiagen). The quality and quantity of RNA were determined using a spectrophotometer. cDNA was synthesized by reverse transcription (High-Capacity cDNA Reverse Transcription Kit, catalog 4368814; Applied Biosystems) with 1 μg of RNA. Quantitative PCR was done with SYBR green dye (Power SYBR Green PCR Master Mix, catalog 4368577; Applied Biosystems) on a real-time thermocycler (QuantStudio 6 Flex Real-Time PCR System; Thermo Fisher Scientific) using a 384-well plate format. Transcripts were amplified using the following DNA oligonucleotides: for *Fap*, forward: 5′-GTCACCTGATCGGCAATTTGT-3′ and reverse: 5′-CCCCATTCTGAAGGTCGTAGAT-3′ normalized to TATA-box binding protein *Tbp*: forward: 5′-GGGTATCTGCTGGCGGTTT-3′ and reverse: 5′-TGAAATAGTGATGCTGGGCACT-3′ and quantified using the comparative CT method (71).

### Data and code availability

Deidentified, individual-level, aptamer-based proteomics and phenotypic data that support the findings from this study have been deposited in the MoTrPAC DataHub and are available as of the date of publication at https://motrpac-data.org/related-studies/heritage-proteomics Overlapping aptamer-based and Ab-based proteomics data in HERITAGE samples are included in the Supplemental Table 2. The study did not generate any unique code. Any additional information required to reanalyze the data reported in this paper is available from the lead contact upon request.

### Statistics

Baseline clinical characteristics of participants are reported as mean ± SD, proportions, or median (IQR), with 2-sample Student’s *t* test, χ^2^ test, and Mann-Whitney U test used to compare differences, respectively. We compared quartiles of BW and composition among protein levels using ANOVA.

Paired *t* tests on logarithmically transformed protein levels were performed to assess changes among individual HERITAGE participants after visual inspection of normal distributions. We used the Benjamini–Hochberg procedure to correct for multiple comparisons. Linear regression was performed to determine the relationship between protein values (both baseline and changes after exercise training [change in protein; posttraining minus pretraining protein] and VO_2_max traits [both baseline and ΔVO_2_max, mL/min]). Protein levels were standardized to mean = 0 and multiples of 1 SD. Spearman’s or Pearson correlations were performed between protein assays and clinical traits according to the presence or absence of normally distributed values. Pathway enrichment analysis was performed using the hypergeometric test implemented in g:profiler using all quantified proteins as the background set (72). GSEA was performed using GSEA Preranked implemented in GSEA, version 4.0.3, and visualized using Cytoscape, version 3.8.2, and the EnrichmentMap tool (26). Unpaired *t* tests were used to test differences in mRNA expression between sedentary and exercising mice. Mortality rates were estimated using the Kaplan–Meier method. In FHS, MDCS, and JHS, the risk of death was quantified using multivariable Cox proportional hazards regression to estimate HRs of plate-adjusted residuals of FAP values after logarithmic transformation, standardization by batch, and rank normalization across a batch. All statistical analyses were performed using either SAS 9.4 (SAS Institute) or R, version 4.1.2 (R Core Team, R Foundation for Statistical Computing).

### Study approval

Written informed consent was provided for all human studies (HERITAGE Family Study, Framingham and Jackson Heart Studies, Malmö Diet and Cancer Study, and Queen’s Exercise Study), and all analyses were approved by the IRB at Beth Israel Deaconess Medical Center. Mouse studies were performed in accordance with the institutional guidelines of The Rockefeller University IACUC (protocol no. 18016-H).

## Author contributions

JMR, PR, MAS, and REG conceived of the study. JMR, CB, MAS, and REG contributed to the methodology. RR contributed to validation. JMR, PR, SD, MJK, PMJB, FM, and BP conducted the formal analysis. FM, PC, and CB contributed to the investigation. AC, JGW, JGS, PC, RR, CB, MAS, and REG contributed resources. JMR, PR, and REG wrote the original draft of the manuscript. JMR, PR, UAT, DHK, JLB, YG, AC, JGW, CB, MAS, and REG reviewed and edited the manuscript. JMR and REG supervised the study. JMR, AC, JGS, RR, CB, MAS, and REG acquired funding for the study.

## Supplementary Material

Supplemental data

Supplemental tables 1-10

## Figures and Tables

**Table 1:**
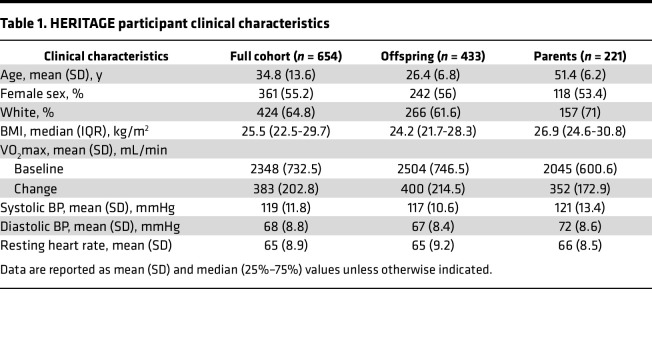
HERITAGE participant clinical characteristics

**Table 2:**
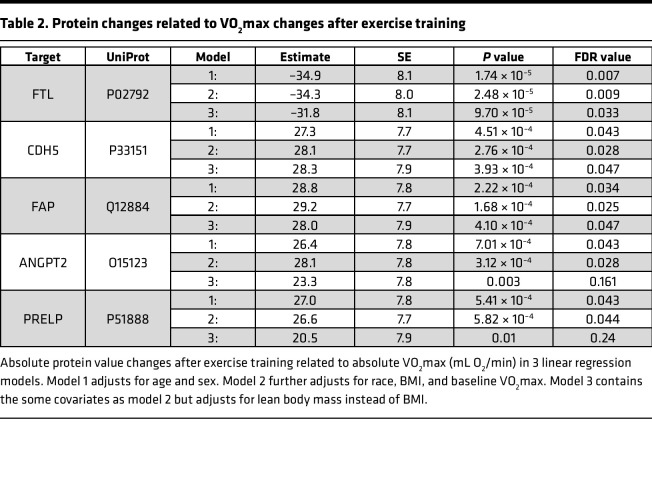
Protein changes related to VO_2_max changes after exercise training

**Table 3:**
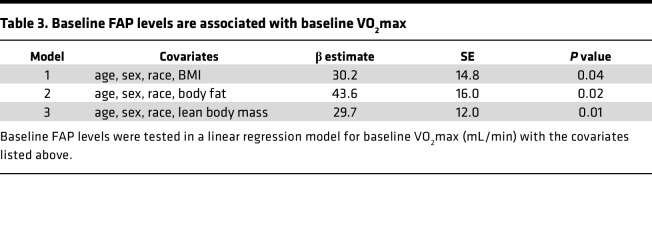
Baseline FAP levels are associated with baseline VO_2_max

## Version 1 04/10/2023

Electronic publication
