# Supplementary material for: Plasma proteomic changes in response to exercise training are associated with cardiorespiratory fitness adaptations
Source: JCI Insight. 2023 Apr 10;8(7):e165867. doi: 10.1172/jci.insight.165867 (PMC10132160; doi:10.1172/jci.insight.165867)
Supplement: Supplemental data [file jciinsight-8-165867-s244.pdf]

Supplemental Table 1. Plasma proteomic changes after 20 weeks of endurance exercise training.

Supplemental Table 2. Spearman correlations between aptamer-based (Somalogic) and antibody-based (Olink) platform for top 25 exercise-responsive proteins.

Supplemental Table 3. cis protein quantitative trait loci (pQTLs) to support aptamer specificity.

Supplemental Table 4. Body weight and composition changes at baseline and after exercise training

Supplemental Table 5. Correlations between protein changes and body weight/composition changes after exercise training

Supplemental Table 6. Pathway enrichment analysis using GO hierarchy among proteins that change with exercise training.

Supplemental Table 7. Clinical characteristics of Validation cohort

Supplemental Table 8. Comparison of HERITAGE and Validation Cohort protein findings, Olink correlations, and proteo-genetic information

Supplemental Table 9. Plasma proteomic associations with FAP in the HERITAGE Family Study.

Supplemental Table 10. Framingham Heart Study, Malmo Diet and Cancer Study, and Jackson Heart Study clinical characteristics.

Supplemental Figure 1. Subcellular location annotations for proteins that changed after exercise training

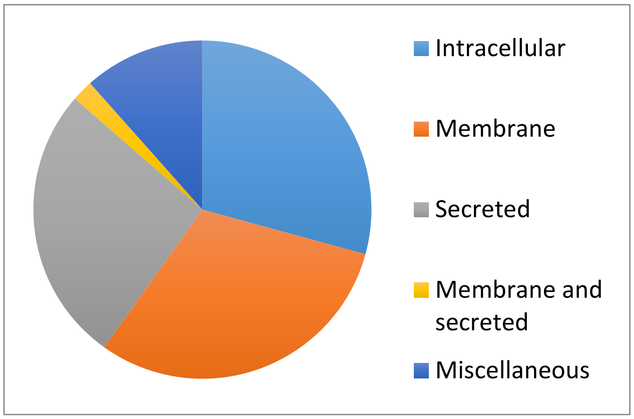

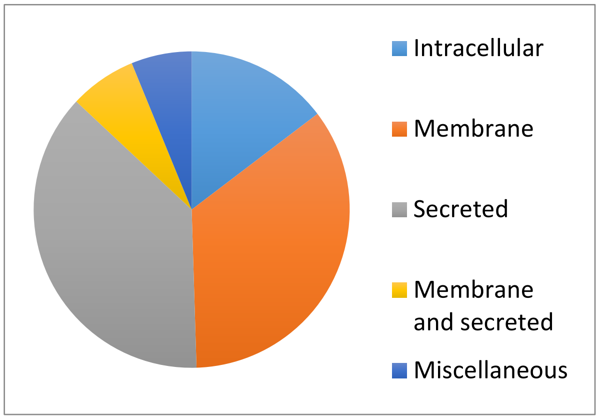

**Proteins that decrease after ET**	N = 147
Intracellular	43
Membrane	45
Secreted	39
Membrane and secreted	3
Miscellaneous	17

**Proteins that increase after ET**	N = 306
Intracellular	45
Membrane	107
Secreted	115
Membrane and secreted	21
Miscellaneous	18

Protein annotations according to the Universal Protein (UniProt) Resource consortium.[1]

Supplemental Figure 2. Gene Set Enrichment Analysis for protein changes associated with VO_2max changes in response to exercise training

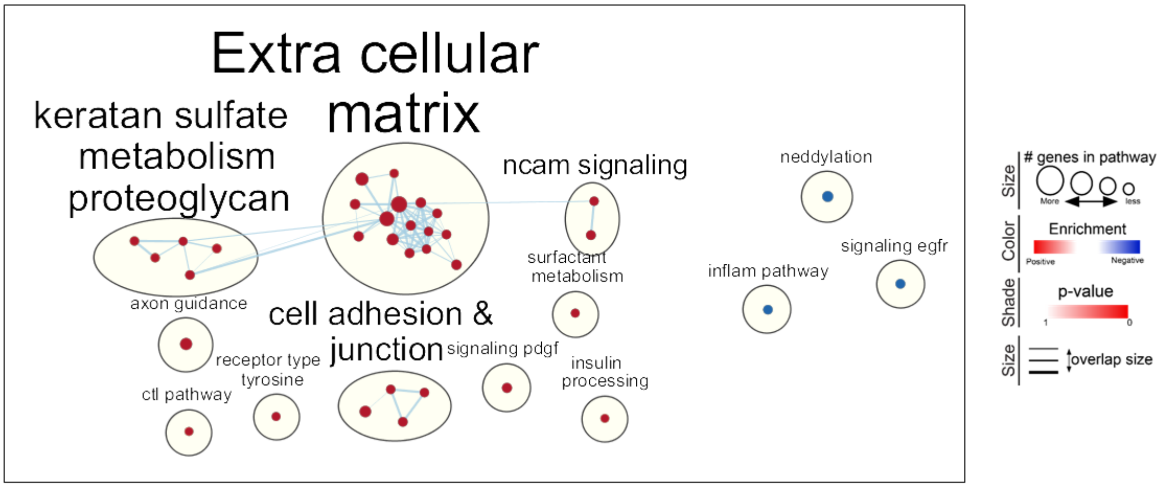

Overview of overrepresented biological pathways and their connectivity. (**A**) Network visualization of GSEA results using the complete dataset of ∆protein-∆VO2max associations. Nodes indicate pathways and edges indicate large gene overlap across pathways. Red dots indicate positive relationships and blue dots indicate negative relationships. Larger circle size connotes a larger number of genes in a pathway and darker shades indicate a higher degree of enrichment. Clusters indicate biological pathways with shared proteins and biological function.

Supplemental Figure 3. Body mass and composition according to plasma FAP levels

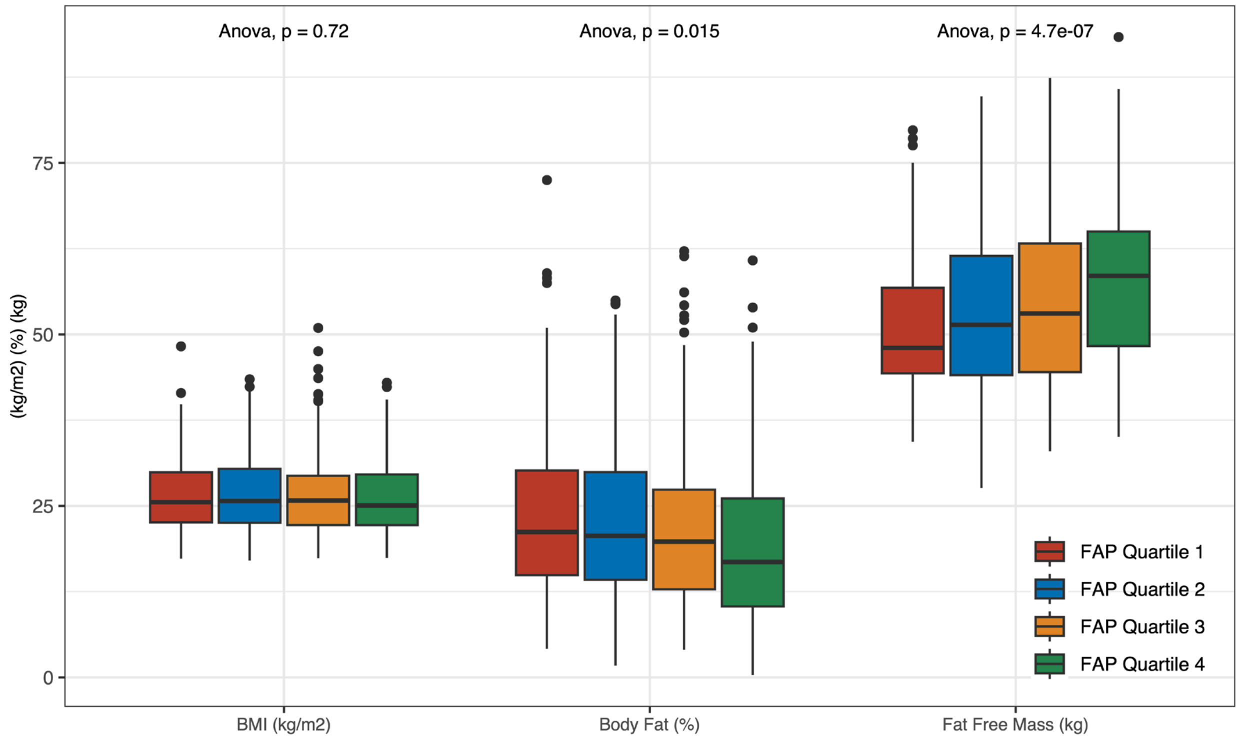

Baseline body mass index, body fat percentage, and fat free mass according to quartiles of baseline FAP level. Boxes represent the 25th and 75th percentile, lines inside represent medians, whiskers represent the upper and lower adjacent values (3/2 times the IQR from the end of the box) as defined by Tukey, and dots represent outliers outside of the whiskers. P-value for ANOVA across FAP quartiles for each phenotype.

References

1. Consortium U. UniProt: a worldwide hub of protein knowledge. *Nucleic Acids Res*. Jan 2019;47(D1):D506-D515. doi:10.1093/nar/gky1049

2. Szklarczyk D, Franceschini A, Wyder S, et al. STRING v10: protein-protein interaction networks, integrated over the tree of life. *Nucleic Acids Res*. Jan 2015;43(Database issue):D447-52. doi:10.1093/nar/gku1003
